# Synthetic Materials that Affect the Extracellular Matrix via Cellular Metabolism and Responses to a Metabolic State

**DOI:** 10.3389/fbioe.2021.742132

**Published:** 2021-10-11

**Authors:** Mireille M.J.P.E. Sthijns, Clemens A. van Blitterswijk, Vanessa L.S. LaPointe

**Affiliations:** ^1^ Department of Cell Biology–Inspired Tissue Engineering, MERLN Institute for Technology-Inspired Regenerative Medicine, Maastricht University, Maastricht, Netherlands; ^2^ Department of Food Innovation and Health at the Centre of Healthy Eating and Food Innovation, Maastricht University, Maastricht, Netherlands

**Keywords:** extracellar matrix, metabolism, materials, regenerative medicine, tissue engineering

## Abstract

In regenerative medicine and tissue engineering, many materials are developed to mimic the extracellular matrix (ECM). However, these ECM-mimicking materials do not yet completely recapitulate the diversity and complexity of biological tissue-specific ECM. In this review, an alternative strategy is proposed to generate ECM, namely synthesizing a material that functions as a drug delivery system, releasing molecules that target cellular metabolic pathways and thereby stimulate the local cells to create their own ECM. This is based on the fact that ECM synthesis, modification, composition, signaling, stiffness, and degradation are modulated by cellular metabolism. Metabolism can be targeted at different levels, ranging from modulating the availability of substrates or co-factors to regulating the activity of essential transcription factors. Depending on the drug of interest, its characteristics, mechanism of action, cellular target, and application, a different drug delivery system should be designed. Metabolic drugs modulating the ECM require cellular uptake for their function, therefore reversible linkers are recommended. Preferably the metabolic modulators are only released when needed, which will be upon a specific metabolic state, a change in ECM stiffness, or ECM remodeling. Therefore, reversible linkers that respond to an environmental stimulus could be incorporated. All in all, a novel strategy is suggested to develop a tissue-specific ECM by generating a synthetic material that releases metabolic molecules modulating the ECM. Various ways to modulate the ECM properties via the metabolism are reviewed and guidelines for the development of these materials are provided.

## Introduction

In regenerative medicine and tissue engineering, a variety of approaches have been taken to engineer the extracellular matrix (ECM) using synthetic or natural building blocks. The aim of these efforts is to generate a matrix that closely mimics the tissue-specific matrix properties including its stiffness, strength, dynamicity, and cell–matrix interactions to enhance cell behavior specific to the tissue of interest (Geckil et al., 2010; Hong et al., 2011; Burdick and Murphy, 2012; Lam et al., 2014).

Despite significant advances in engineering the ECM, synthetic materials have yet to fully capture its diversity and complexity (Kyburz and Anseth, 2015). Some scientists have turned to using biological tissue as a source of ECM proteins. For example, following decellularization, these proteins can be used as a scaffold or as a bioink for 3D printing (Uriel et al., 2009; Dzobo et al., 2019; Fernández-Pérez and Ahearne, 2019). Other approaches have embraced a combined synthetic and natural approach, for example by coating synthetic materials with biological motifs to enhance cell–matrix interactions. The best known example of this approach is the addition of an RGD peptide to a scaffold, which cells can use for integrin-mediated adhesion and subsequent signaling (Ingavle et al., 2014; Xing et al., 2020). However, while these approaches may capture some of the important features of the ECM, it remains challenging to produce tissue-specific ECM with the correct biological (e.g., expression of specific ECM proteins and post-translational modifications) and physical (e.g., stiffness, viscoelasticity) properties. Many approaches are also hindered by additional disadvantages such as the lack of reproducibility and tunability.

Here, an alternative strategy for generating tissue-specific ECM is reviewed. In this strategy, instead of directly engineering an ECM, synthetic materials can be used to modulate cellular metabolism and thus stimulate cells to synthesize and remodel their own ECM (Ma et al., 2019). A synthetic material is chosen for this strategy because it offers a blank-slate, whereas a naturally-derived ECM can possess tissue-specific cues, which is seen in examples where it induces tissue-specific differentiation (da Mata Martins et al., 2020; Parmaksiz et al., 2020). From a synthetic material, the ECM stiffness can be tuned and bioactive molecules can easily be incorporated (Kyburz and Anseth, 2015). The synthetic material therefore functions as a drug delivery system to target metabolic pathways in the cells (Tiwari et al., 2012; Tibbitt et al., 2016; Li et al., 2019), which also offers the benefit of local drug delivery to enhance the therapeutic effect (Hwang et al., 2020). These metabolic pathways leading to changes in ECM production and remodeling are consistently expressed in all different cell types and can be modulated on demand. In this mini-review, the mechanisms by which cellular metabolism affects the ECM, the various ways to modulate the ECM by targeting the metabolism, and guidelines for the development of materials that employ this approach are reviewed.

## Cellular Metabolism Affects the ECM Synthesis, Composition, and Physical Properties

Every cell needs energy to perform its function. They are therefore equipped with various metabolic pathways to generate energy from substrates ranging from glucose to lipids. These metabolic pathways are regulated based on the available substrates and oxygen levels. Some pathways generate more energy (e.g., oxidative phosphorylation, the aerobic oxygen-dependent pathway) than others (glycolysis, the anaerobic oxygen-independent pathway).

Every tissue has a specific ECM in terms of its structure and composition (Frantz et al., 2010). Because ECM synthesis requires a lot of energy, its synthesis and the composition of the resulting matrix can be affected by the metabolism. Furthermore, modulating the cellular metabolism can change the physical properties of the ECM, such as its stiffness and degradation. All of these naturally occurring metabolic pathways that affect ECM properties can be targets of a synthetic materials-based approach to generate tissue-specific ECM (Romani et al., 2021).

### Different ECM Components are Expressed due to Changes in Metabolism

Different metabolic targets exist to modulate ECM expression. In general, when there is an excess of nutrients, AMP-activated protein kinase (AMPK) is decreased and mammalian target of rapamycin complex 1 (mTORC1) is increased, which subsequently increases fibronectin assembly and integrin activation (Table 1). AMPK has also been shown to regulate hyaluronan synthesis by phosphorylating and inhibiting hyaluronan synthase 2 (Xing et al., 2020). In addition, another in fibroblasts and epithelial cells from the lung, kidney, liver, and skin, it has been shown that the Sterol regulatory element-binding proteins (SREBP)/mevalonate metabolism regulates the Yes-associated protein 1 (YAP1)/transcriptional coactivator with PDZ-binding motif (TAZ) signaling (Sorrentino et al., 2014; Noguchi et al., 2018). This activation of YAP/TAZ signaling induces the synthesis of (profibrotic) ECM proteins like collagen I (Table 1). Conversely, when nutrients are in short supply, fibronectin assembly is decreased and integrin internalization and ECM degradation are subsequently increased (Dornier and Norman, 2017).

**TABLE 1:** Metabolic pathways regulating ECM expression.

Metabolic target	ECM expression	References
AMP-activated protein kinase (AMPK)	Fibronectin increase	Xing et al. (2020)
Sterol regulatory element-binding proteins (SREBP)	Collagen I increase	(Sorrentino et al., 2014; Noguchi et al., 2018)
Peroxisome proliferator-activated receptor gamma (PPARγ)	Collagen VI and thrombospondin 1 increase	Okuno et al. (2002)

This relationship between nutrients and ECM also exists in differentiating stem cells. For example, during adipocyte differentiation, cellular metabolism changes due to the uptake and storage of fatty acids (Mariman and Wang, 2010). This change affects the ECM composition through peroxisome proliferator-activated receptor gamma (PPARγ), a key transcription factor in adipocyte differentiation, which upregulates the synthesis ECM components collagen VI and thrombospondin 1 (Okuno et al., 2002; Table 1). All in all, the energetic substrate leads to metabolic changes that can affect the expression of ECM proteins like fibronectin or collagen.

### Hypoxia Induces a Stiffer Matrix

Another means by which the ECM can be modulated is through oxygen availability, which can also affect the induction of metabolic pathways, leading to mechanical modulation of the ECM. In hypoxia, which is a low level of oxygen, more anaerobic metabolic pathways are upregulated, and the expression of collagen I is reduced while the expression of collagen IV is increased (Tajima et al., 2001; McKay et al., 2017). However, it is not just the ECM synthesis and composition that are affected by metabolism; the physical properties of the ECM are also influenced. When the metabolism of chondrocytes is affected by hypoxia and there is a subsequent diminished glucose oxidation, the collagen synthesis is also reduced (Stegen et al., 2019). As the cells switch from a metabolism based on glucose and fatty acids to glutamine, the enhanced α-ketoglutarate formation from glutamine leads to increased proline and lysine hydroxylation of collagen (Stegen et al., 2019; Table 2). This hydroxylation of collagen increases ECM stiffness and prevents matrix degradation. As another example, in hepatic stellate spheroids, the addition of the glycolytic metabolite phosphoenolpyruvate has been shown to increase α-smooth muscle actin, thereby stiffening the matrix (Fujisawa et al., 2020; Table 2).

**TABLE 2:** Metabolic pathways regulating ECM stiffness.

Metabolic target	ECM	Mechanical result	References
α-ketoglutarate formation from glutamine	Proline and lysine hydroxylation of collagen	Stiffer matrix	Stegen et al. (2019)
Phosphoenolpyruvate	α-smooth muscle actin increase	Stiffer matrix	Fujisawa et al. (2020)
Peroxisome proliferator-activated receptor gamma (PPARγ)	Matrix metalloproteinase-1 expression	Softer matrix	(François et al., 2004; Mariman and Wang, 2010; Taddese et al., 2010)
Pyruvate	Prolyl-4-hydroxylase activation	Stiffer matrix	Elia et al. (2019)

Metabolism can also affect the degradability of the ECM through, for example, matrix metalloproteinases (MMPs). Briefly, MMPs can be classified in specific groups, one of which are the collagenases. These MMPs cleave collagen at specific sites, except when the collagen is hydroxylated at a nearby proline or lysine residue (Taddese et al., 2010). An example of how metabolism affects the degradability of the ECM is the activation of PPARγ, which increases the expression of MMP-1 (François et al., 2004; Table 2). Similar relationships are seen in medicine, where obesity leads to adipocyte hypertrophy and overgrowth, leading to hypoxia and decreased energy production via the tricarboxylic acid cycle. This inhibits proline hydroxylation, which increases ECM degradation by MMPs and leads to ECM instability (Mariman and Wang, 2010). Finally, in breast cancer cells, it has been shown that pyruvate metabolism induces collagen hydroxylation by activating prolyl-4-hydroxylase, which decreases MMP8 degradation and subsequently decreases metastatic growth of the cancer cells (Elia et al., 2019; Table 2). Overall, due to low oxygen, alternative metabolic pathways are induced that affect ECM crosslinking or release enzymes that are involved in ECM remodeling affecting its mechanical properties.

### Other Interactions Between Cellular Metabolism and ECM Properties

It is also interesting to note that in addition to the cellular metabolism determining ECM properties, it is also the case that the ECM conversely affects cellular metabolism. However, this is outside of the scope of this mini-review and has been extensively described elsewhere (Mah et al., 2018; Fernie et al., 2020). For example, when cells detach from the ECM that leads to decreased glucose uptake, glycolysis and oxidative phosphorylation (Mason et al., 2017). In addition, a low level of hyaluronan leads to diminished internalization of the glucose transporter GLUT1 that subsequently decreases all glucose-related cellular metabolism (Sullivan et al., 2018). Decreased hyaluronan activates receptor tyrosine kinase (RTK), which subsequently induces ZFP36. ZFP36 leads to thioredoxin interacting protein (TXNIP) degradation and decreases GLUT1 internalization (Katsu-Jiménez et al., 2019). ECM detachment of ECM expression can both affect substrate uptake or increase metabolism.

In summary, cellular metabolism can affect the ECM synthesis, modification, composition, signaling, stiffness, and degradation. In addition, the ECM can also influence the cellular metabolism. These studies indicate interesting metabolic targets that can be used to modulate the ECM. Next, we will describe how (bio)materials scientists and tissue engineers can harness this information.

## A Toolbox for (Bio)material Scientists to Influence Metabolism and Thereby Affect ECM

Methods to modulation the ECM by influencing cellular metabolism can be incorporated in the design of materials or scaffolds for tissue engineering. Material scientists have previously developed various effective tools for drug delivery that can be applied (Li et al., 2019). Depending on the drug of interest, its characteristics, mechanism of action, cellular target and application, different materials, and strategies to modify the material can be chosen. ECM-like hybrid materials are very suitable for the addition of a drug-delivery element and application for regenerative medicine purposes (Hinderer et al., 2016; Setayeshmehr et al., 2019; Zhu et al., 2019). All in all, synthetic materials could be combined with a drug delivery element depending on the drug of choice.

Different metabolic drugs exist to target different metabolic pathways. A metabolic pathway can be targeted at different levels, for example an essential co-factor can be scavenged or an inhibitory analog of an essential substrate with higher affinity can be provided (Park et al., 2008; Zhdanov et al., 2015; Zhang et al., 2016). Furthermore, a transcription factor can be inhibited directly by keeping it in its inactive form (Gu et al., 2016). We will first discuss the selection of a drug, which will be followed by an overview of the delivery strategies that (bio)materials scientists can employ.

### Drugs can Target Metabolism by Affecting Essential Co-Factors or Metabolic Transcription Factors

There are many drugs that can target metabolism, for example, by targeting a co-factor for a metabolic pathway or directly targeting a transcription factor. Examples of drugs that target an essential co-factor for a metabolic pathway are deferoxamine (DFO) and dimethyloxalylglycine (DMOG), which both affect the metabolic transcription factor HIF1α. DFO is an iron chelator, while DMOG is a synthetic analogue of α-ketoglutarate. Both iron and α-ketoglutarate are essential substrates for prolyl-4-hydroxylase, which hydroxylates HIF1α in normoxia to target HIF1α for proteosomal degradation (Iommarini et al., 2017; Donneys et al., 2019). However, during hypoxia or when adding DFO or the competitive inhibitor DMOG, prolyl-hydroxylases do not hydroxylate HIF1α. This leads to HIF1α-mediated signaling, but also decreases proline and lysine hydroxylation of collagen, which decreases matrix stiffness and leads to a higher sensitivity of the matrix for degradation (Rappu et al., 2019; Stegen et al., 2019).

An example of a metabolic drug that directly targets a metabolic transcription factor is the inhibitor AS1842856 that inhibits FOXO1 by binding to its active non-phosphorylated form. FOXO1 mediates the activation of Glucose 6-phosphatase and inhibits glucokinase. In hepatocytes, insulin represses glucose production and enhances lipogenesis by inhibiting FOXO1 activation (Langlet et al., 2017). Depending on the metabolic pathway that needs to be activated, a different metabolic drug can be chosen.

### Criteria for Metabolic Drugs are Mechanism of Action, No Therapeutic Toxicity and Potential for Clinical Use

In this strategy, it is important that the characteristics of the drug of interest be taken into account. There are numerous considerations that should be given to selecting a drug for the purpose of modulating cellular metabolism and thereafter the ECM. Firstly, it is essential that the drug does not have any toxic effects on the cells at its effective concentration and exposure time (Suzman et al., 2019; Hoy, 2020). Preferably, the drug is FDA/EMA-approved like DFO, which has been approved for iron overload (Kontoghiorghes and Kontoghiorghe, 2016; Sun et al., 2020; Xu et al., 2020; Takpradit et al., 2021). After selecting the drug of interest based on characteristics, mechanism of action and cellular target, the next step will be to include it in the right material and apply the right release strategy.

### The Mechanism of Action of the Metabolic Drug Determines Which Material and Coupling Strategy will be Chosen.

The physical properties of the tissue of interest is important for the choice of the material, while the mechanism of action of the metabolism-modulating drug defines the coupling strategy. For example, depending on the stiffness of the ECM of the target tissue, a different material could be selected, ranging from solid materials to soft hydrogels. PEG, PVA, and PHEMA are frequently used for engineering the ECM, and they can be co-polymerized with different polymers to tune the physical properties (Unal and West, 2020), which can also be an advantage in 3D printing applications (Da Silva et al., 2020). For an optimal mechanical strength, dynamic viscoelastic hydrogels are of particular interest because of the tunability of their stiffness and their resemblance to the ECM (Hafeez et al., 2018; Table 3).

**TABLE 3:** Design criteria for synthetic materials that affect the extracellular matrix via cellular metabolism.

Biological criterium	Material design choice	Reason	References
Tissue stiffness	Dynamic viscoelastic hydrogels	Tunability	Hafeez et al. (2018)
Mechanism of action drug	a) Reversible linker	a) Transmembrane transport essential	(Schreier et al., 2000; Elvira et al., 2005)
a) Intracellular target	b) Nonreversible linker	b) Extracellular binding sufficient	
b) Extracellular target			
Stimulus or time-responsive effects	Intelligent materials	Release based on environmental stimulus	(Rao N et al., 2012; Liang and Liu, 2016; Tao and He, 2018; Unal and West, 2020)

The drug coupling or delivery method needs to be chosen based on the drug of interest and its cellular target (Table 3). For example, if the metabolic drug needs to be released to be functional or taken up by the cell to induce its biological effect, it is essential to include a reversible linker into the material design. Biodegradable linkers can be used, including: carbonates, anhydrides, esters, urethanes, amides, or orthoesters (Elvira et al., 2005). If the drug of interest is of amphiphilic or hydrophobic nature, it can self-associate in surfactant materials (Schreier et al., 2000). In order to control drug release, linkers that depend on the environmental stimulus could be applied or designed to modulate the timing of drug release (Table 3).

For some metabolic modulators that regulate the ECM, a more sustained release is required. Many strategies are available to induce a sustained release, ranging from coupling, host-guest chemistry to encapsulating in cellulose capsules (Bakker et al., 2018a; Bakker et al., 2018b; Sun et al., 2019). Furthermore, many different intelligent biomaterials have been developed that respond to the environment, including cues such as pH, reactive oxygen species (ROS), MMPs, or even mechanotransduction. Depending on the degradation rate by MMPs, a different polymer can be chosen (Unal and West, 2020). An example of how environmental cues can be used is in the setting of an engineered tissue that lacks vasculature. This will lead to hypoxia and enhanced glycolysis and lactic acidosis in the surrounding tissue that decreases the pH. This pH decrease can be used to prevent associated changes in the surrounding ECM by releasing a drug that influences the metabolism and subsequent ECM remodeling. A hydrazine linker is a commonly used pH-inducible release linker, since it is cleaved in an acidic environment (Rao N et al., 2012). Similarly, the decreased oxidative phosphorylation present in hypoxia also leads to incomplete mitochondrial respiration and the formation of ROS. These ROS could be another stimulus that releases metabolic drugs influencing ECM in response to metabolic changes. ROS can reduce disulfide bonds inducing the release of the coupled drug molecule. Next to the disulfide bonds, there exist many different ROS or redox-responsive linkers (Liang and Liu, 2016; Tao and He, 2018).

In summary, the tissue of interest defines the material choice. Depending on the local ECM stiffness and presence of local cells, more or less metabolic modulator will be released, making synthesis and stiffness of the ECM tissue-specific. In addition, depending on the characteristics of the metabolic drug modulating ECM and the local ECM, different drug delivery strategies should be used. Because most metabolic drugs that affect the ECM require cellular uptake for their function, reversible linkers should be applied. Intelligent materials that respond to the pH, ROS, MMPs, or mechanical signals from the environment enhance the biological application of these materials.

## Discussion

**FIGURE 1:**
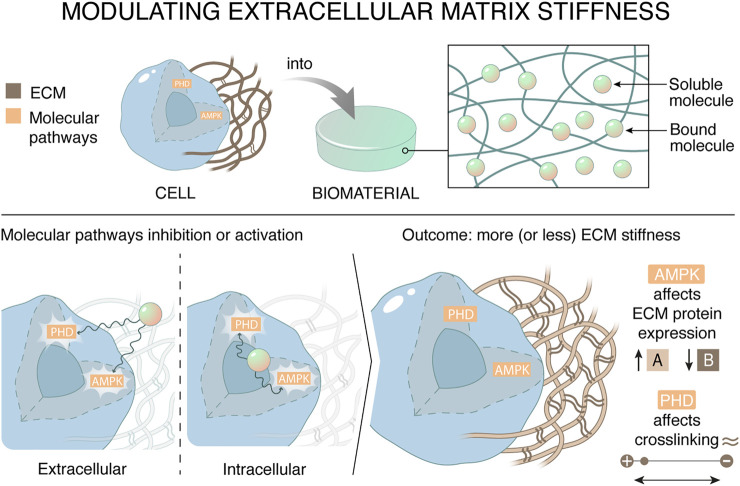
Materials can affect the stiffness of the extracellular matrix by regulating cellular metabolism. The synthetic drug delivery system targets metabolic mediators AMP-activated protein kinase (AMPK) and prolyl hydroxylases in tissue resident cells, which in their turn decreases ECM stiffness. There are two ways to regulate the outcome of ECM stiffness, namely by affecting ECM expression or by affecting ECM crosslinking. This can be achieved by targeting metabolic pathways by scavenging essential cofactors extracellularly or directly inhibiting metabolic targets.

To promote both the formation of a naturally complex ECM, while preventing the variability in production, studies have shown the potential of a synthetic material that functions as a drug delivery system to release metabolic modulators that influence the synthesis and modification of ECM in resident cells (Figure 1).

AMPK, PPARγ, SREBP, or prolyl hydroxylases are metabolic targets known to influence the composition and/or stiffness of the ECM. Different metabolic drugs exist that target metabolic pathways and thereby influence the ECM synthesis and/or modification. The metabolic drugs can target co-factors or substrates or interact with a metabolic transcription factor. The characteristics, mechanism of action, cellular target and application of the drug of interest inform the choices for the synthetic materials and the drug-coupling strategy. If the metabolic molecule needs to bind the intracellular enzymes or transcription factors for performing its function, a reversible linker could be used. These reversible linkers could respond to metabolic (pH, ROS) or ECM-related (MMP-mediated degradation or mechanical forces) microenvironmental stimuli.

In conclusion, we have described the potential of a synthetic material with a drug-delivery element to modulate cellular metabolism and thereby regulate the ECM. This strategy confers reproducibility in production while capturing the complexity of the tissue-specific ECM.

In the future, multidrug spatiotemporal controlled hydrogels could contribute to induce a tunable time or concentration-specific drug release. This is especially important because biological systems are complex with multiple (negative) feedback loops. During physiological metabolism, a metabolic pathway is activated, which leads to a new cue influencing the next pathway. This could be the inspiration for the next step in material engineering. Multidrug-responsive hydrogels could be designed that contain multiple drug delivery elements that are also capable of responding to a second cellular mediators, also called intelligent multicommunicating materials (Brown and Anseth, 2017; Leijten et al., 2017). These multidrug spatiotemporal controlled hydrogels could be the next generation of cell–material communication.
